# Variants in Miro1 Cause Alterations of ER-Mitochondria Contact Sites in Fibroblasts from Parkinson’s Disease Patients

**DOI:** 10.3390/jcm8122226

**Published:** 2019-12-16

**Authors:** Clara Berenguer-Escuder, Dajana Grossmann, Franҫois Massart, Paul Antony, Lena F. Burbulla, Enrico Glaab, Sophie Imhoff, Joanne Trinh, Philip Seibler, Anne Grünewald, Rejko Krüger

**Affiliations:** 1 Luxembourg Centre for Systems Biomedicine (LCSB), University of Luxembourg, 4367 Belvaux, Luxembourg; dajana.grossmann@uni.lu (D.G.); francois.massart@uni.lu (F.M.); paul.antony@uni.lu (P.A.); enrico.glaab@uni.lu (E.G.); anne.gruenewald@uni.lu (A.G.); 2 Department of Neurology, Northwestern University Feinberg School of Medicine, Chicago, IL 60611, USA; lena.burbulla@northwestern.edu; 3 Institute of Neurogenetics, University of Lübeck, 23562 Lübeck, Germany; sophie.imhoff@online.de (S.I.); joanne.trinh@neuro.uni-luebeck.de (J.T.); philip.seibler@neuro.uni-luebeck.de (P.S.); 4 Luxembourg Institute of Health (LIH), 1445 Strassen, Luxembourg; 5 Parkinson Research Clinic, Centre Hospitalier de Luxembourg (CHL), 1460 Luxembourg, Luxembourg

**Keywords:** Parkinson´s disease, mitochondria-ER contact sites, Miro1

## Abstract

Background: Although most cases of Parkinson´s disease (PD) are idiopathic with unknown cause, an increasing number of genes and genetic risk factors have been discovered that play a role in PD pathogenesis. Many of the PD-associated proteins are involved in mitochondrial quality control, e.g., PINK1, Parkin, and LRRK2, which were recently identified as regulators of mitochondrial-endoplasmic reticulum (ER) contact sites (MERCs) linking mitochondrial homeostasis to intracellular calcium handling. In this context, Miro1 is increasingly recognized to play a role in PD pathology. Recently, we identified the first PD patients carrying mutations in *RHOT1*, the gene coding for Miro1. Here, we describe two novel *RHOT1* mutations identified in two PD patients and the characterization of the cellular phenotypes. Methods: Using whole exome sequencing we identified two PD patients carrying heterozygous mutations leading to the amino acid exchanges T351A and T610A in Miro1. We analyzed calcium homeostasis and MERCs in detail by live cell imaging and immunocytochemistry in patient-derived fibroblasts. Results: We show that fibroblasts expressing mutant T351A or T610A Miro1 display impaired calcium homeostasis and a reduced amount of MERCs. All fibroblast lines from patients with pathogenic variants in Miro1, revealed alterations of the structure of MERCs. Conclusion: Our data suggest that Miro1 is important for the regulation of the structure and function of MERCs. Moreover, our study supports the role of MERCs in the pathogenesis of PD and further establishes variants in *RHOT1* as rare genetic risk factors for neurodegeneration.

## 1. Introduction

Parkinson’s disease (PD) is a complex neurodegenerative disorder with a largely unknown molecular pathogenesis. Although most PD cases are classified as idiopathic with unknown cause, a growing number of genes and genetic risk factors have been identified, which contribute to the development of PD [1] Many of these genes were found to be involved in common pathways suggesting that, at least in a subgroup of cases, PD might be caused by similar pathological mechanisms. PD-associated mutations in PINK1, Parkin and LRRK2 for example cause impairments of mitochondrial quality control and subsequent mitochondrial dysfunction [2] Mitochondrial-endoplasmic reticulum (ER) contact sites (MERCs) are important connections required for the proper function of mitochondria, i.e., by facilitating the exchange of metabolites, calcium and lipids between both organelles [3,4], thereby maintaining mitochondrial homeostasis. PINK1, Parkin, LRRK2 and α-synuclein (α-syn) were recently found to be involved in the regulation of MERCs. Impaired function of these proteins caused fragmentation of the mitochondrial network, mitochondrial calcium dyshomeostasis and alterations of the amount of MERCs [5,6,7].

Interestingly, Miro1 plays a central role in a number of molecular and organellar pathways, which are affected in PD. Via its interaction with PINK1, Parkin and LRRK2 [2], Miro1 interferes with mitochondrial quality control and MERCs [5,8,9]. Having access to fibroblasts from PD patients carrying different Miro1 mutations, we explored the role of Miro1 at MERCs in more detail. In this study, we provide further evidence for a role of rare genetic variants in *RHOT1* in PD, now presenting a wider spectrum of Miro1 point mutations in a total of 4 independent patients. In agreement with our previous findings in Miro1-R272Q and Miro1-R450C mutant fibroblasts [10], fibroblasts with the newly identified heterozygous mutations T351A and T610A also display a reduction of MERCs as well as impaired calcium homeostasis. Furthermore, we analyzed the MERC composition in all four Miro1-mutant cultures and found differences in the recruitment of Miro1 protein to MERCs and in the amounts of different types of MERCs compared to control fibroblasts. Our data further establish mutations in *RHOT1* as risk factor for PD and support recent studies implicating dysfunctional MERCs in the pathogenesis of PD.

## 2. Experimental Section

### 2.1. Identification of Miro1-T351A and T610A Mutations

We studied exome data from 86 subjects (62 with PD and 24 controls). The ‘convenient patient sample’ was comprised of early-onset PD cases. The patients were not known to carry either a pathogenic or likely pathogenic genetic variant in any known PD gene. However, they carried heterozygous variants in Miro1 (T351A and T610A). All patients were examined and diagnosed by movement disorder specialists. The patient harboring the T351A had an age of onset of 60 years and was 65 years old, when the skin biopsy was taken. He suffered from resting tremor, bradykinesia as well as restless legs syndrome. He was treated with a dopamine agonist at the time of examination. The patient with the T610A mutation developed PD at age 40 and was sampled at age 45. He showed signs of bradykinesia, rigidity and a good response to L-DOPA. Moreover, he suffered from depression.

All participants provided informed consent prior to donating a blood sample for genetic analysis and are from Germany or of other European descent. Local ethics approval was obtained from the Research Ethics Board of the University of Lübeck, Germany.

### 2.2. Exome Sequencing

Exome sequencing was performed with Illumina’s Nextera Rapid Capture Exome Kit followed by massively parallel sequencing on a NextSeq500 Sequencer (Illumina, San Diego, CA, USA). Raw sequencing reads were converted to fastq format using bcl2fastq software (Illumina). Using an in-house developed pipeline for exome data analysis, the reads were aligned to the human reference genome (GRCh37, hg19 build) with burrows-wheeler algorithm (BWA) software and the mem algorithm. Alignments were converted to binary bam file and variant calling was performed using three different variant callers (GATK HaplotypeCaller, freebayes and samtools). Variants were annotated using Annovar and in-house ad-hoc bioinformatic tools. Exome variants were filtered for (1) minor allele frequency (MAF) <0.01 in Miro-1 (*RHOT1*) in Genome Aggregation Database (GnomAD); (2) functional impact: Non-synonymous, stop-gain, frameshift, and splicing variants; and (3) Phred quality score >220, coverage >20× and variant allele fraction > 40% of called reads. All the selected variants identified in two patients were validated by Sanger sequencing as previously described [11]. Primers with the following sequences were used: for the mutation T351A: (forward: TGTGTTTCTTCAGGATAGAGAC; reverse: GATATCAGCAGCTAATCTTGC); for the mutation T610A: (forward: GGGCCACACTGATAGAATAG; reverse: ACGTAATATATAGCTAGGCAGG).

### 2.3. Fibroblast Cell Culture

Fibroblasts were obtained from the identified two male PD patients. Fibroblasts from three age-matched controls were received from the Neuro-Biobank of the University of Tübingen, Germany. In Lübeck as well as in Tübingen, skin biopsies to establish fibroblast cultures are taken from the arm of the individuals. Cell culture conditions and immortalization of native fibroblasts were explained in our previous study [10].

### 2.4. Western Blot Analysis

Immortalized fibroblasts were lysed in RIPA buffer with 1× complete protease inhibitor (Roche, Mannheim, Germany). Sodium dodecyl sulfate polyacrylamide gel electrophoresis and Western blot analysis was performed using antibodies against Miro1 (Sigma Aldrich, Munich, Germany, WH0055288M1), Tom20 (Santa Cruz Biotechnologies, Dallas, TX, USA, sc-17764), Rab9 (Santa Cruz Biotechnologies, Dallas, TX, USA, sc-74482), and β-Actin (Thermo Scientific, Braunschweig, Germany, MA1-744).

### 2.5. Homology Model of Miro1

Homology models for isoform1 of the human Miro1 protein were built using a crystal structure of sub-domains from this protein in the GMPPCP-bound state with a resolution of 2.5 Å (PDB: 5KSZ) as the main template. Since this template only covers the position of the mutated residue T351, but not the position of the second mutated residue T610, two separate models were built: One high-quality model focusing on the region covered by the template (created using the SWISS-MODEL software [12]), and one lower-quality model covering the entire protein sequence (created using a multi-template approach for protein threading, RaptorX [13]). For all analyses and predictions related to the residue T351, the high-quality model was used, whereas all analyses for residue T610 were conducted using the lower-quality model with complete sequence coverage.

Structure visualizations of the Miro1 isoform1 protein were generated with the software Chimera [14], using the domain annotations from the Uniprot database [15]. Next, the transmembrane regions for the protein were predicted by applying the Constrained Consensus Topology approach (software CCTOP [16] with default parameters). Finally, mutation effects were predicted using the structure-based methods SDM, mCSM [17], DUET [18], and the more sequence-based approaches SIFT [19], Polyphen2 [20] (as implemented in the Variant Effect Predictor software, VEP [21]), and SNAP2 [22].

### 2.6. Live Cell Imaging for Calcium Analysis

The protocol for live cell imaging of cytosolic calcium levels using Fluo4-AM staining and treatment with thapsigargin and Ru360 was described in detail before [10]. Live cell imaging for the analysis of calcium-induced fragmentation using MitoTracker green FM staining and ionomycin treatment was likewise described in detail [10]. The aspect ratio was determined to assess mitochondrial fragmentation. In brief, the aspect ratio is calculated by the minor axis of mitochondria divided by the major axis of mitochondria and indicates mitochondria fragmentation. The smaller the value of the aspect ratio, the smaller the mitochondria, indicating higher fragmentation. Mitochondrial morphology was analyzed using ImageJ software. Images were obtained with a Live cell Microscope Axiovert 2000 with spinning disc, plan-aprochromate objectives and Hamamatsu camera C11440 (Carl Zeiss Microimaging GmbH, Jena, Germany) in a humidified atmosphere containing 5% CO_2_ at 37 ℃, using a 20× objective for Fluo4-AM or a 40× objective for MitoTracker green, respectively.

### 2.7. Immunocytochemistry for Analysis of MERCs

For quantification and analysis of MERCs, native fibroblasts were fixed in 4% paraformaldehyde for 15 min for subsequent immunocytochemistry. Cells were then labeled with antibodies against protein disulfide-isomerase (PDI) (2446S, dilution 1:1000, secondary antibody: Goat anti-rabbit Alexa Fluor 488; Cell Signaling Technology, Danvers, MA, USA; A-1000, dilution 1:1000; Life Technologies, Carlsbad, CA, USA), Tom20 (sc-17764, dilution 1:500, secondary antibody: Goat anti-mouse Alexa Fluor 647; Santa Cruz Biotechnologies, Dallas, TX, USA; A-21235, dilution 1:1000; Life Technologies, Carlsbad, CA, USA) and Miro1 (Sigma Aldrich, Munich, Germany, WH0055288M1, 1:1000; secondary antibody: Life Technologies, Carlsbad, CA, USA, goat anti-mouse Alexa Fluor 647 1:1000). Data were analyzed using MATLAB. Images were obtained with a Live cell Microscope Axiovert 2000 with spinning disc, plan-aprochromate objectives and Hamamatsu camera C11440 (Carl Zeiss Microimaging GmbH, Jena, Germany), using a 40× objective. Every field consisted of z-stacks of 0.5 µm interval. Data was analyzed using MATLAB. ER area per cell (in pixel) was quantified from the area covered by the PDI signal and mitochondria area per cell (in pixel) was derived from the area covered by Tom20 signal.

### 2.8. Analysis of Miro1 Localization to Different Cellular Compartments

Immortalized fibroblasts were transfected with mito-GFP using *Trans*IT^®^-2020 transfection reagent (Mirus Bio, Madison, WI, USA, MIR 5400) according to the manufacturer’s protocol. At 24 h after transfection, cells were fixed with 4% PFA for 15 min and stained with antibodies against Calnexin (Cell Signaling Technology, Danvers, MA, USA, C5C9, 1:500; secondary antibody: Life Technologies, goat anti-rabbit Alexa Fluor 568, 1:1000) and Miro1 (Sigma Aldrich, Munich, Germany, WH0055288M1, 1:1000; secondary antibody: Life Technologies, Carlsbad, CA, USA, goat anti-mouse Alexa Fluor 647 1:1000). Images were obtained using a Axiovert 2000 Microscope with spinning disc, plan-apochromate objectives and Hamamatsu camera C11440 (Carl Zeiss Microimaging GmbH, Jena, Germany), 63× objective. Image analysis was performed with MATLAB.

### 2.9. Imaging of ER-Mitochondria Contact Sites Using the SPLICS Method

Immortalized fibroblasts were transfected with the SPLICS-short or the SPLICS-long construct [23], respectively, using *Trans*IT^®^-2020 transfection reagent (Mirus Bio, Madison, WI, USA, MIR 5400). At 12 h after transfection, cells were stained with 0.1 µM MitoTracker deep red FM (Thermo Scientific, Braunschweig, Germany) for 30 min and imaged using a Axiovert 2000 Microscope with spinning disc, plan-apochromate objectives and Hamamatsu camera C11440 (Carl Zeiss Microimaging GmbH, Jena, Germany), 63× objective. Image analysis was performed with MatLab.

### 2.10. Quantification of Co-Localization of Mitochondria and LC3

Immortalized fibroblasts were transiently transfected with mito-DsRed [24] and eGFP-LC3 [25] constructs using *Trans*IT-2020 transfection reagent (Mirus Bio, Madison, WI, USA, MIR 5400). After 24 h, fibroblasts were treated with 25 µM CCCP or 10 nM Bafilomycin A1 for 2 h or 6 h. Co-localization of mitochondria (indicated by mito-DsRed signal) and autophagosomes (indicated by eGFP-LC3 puncta) was considered as mitophagy events.

### 2.11. Analysis of Autophagosome Formation

Immortalized fibroblasts were transfected with mito-dsRed [24] using *Trans*IT-2020 transfection reagent (Mirus Bio, Madison, WI, USA, MIR 5400). After 24 h, autophagosome formation was assessed by staining native fibroblasts with 0.2 mM 18:1 NBD-PS (Sigma Aldrich, Munich, Germany, 810198C) for 30 min at 37 ℃. Cells were subsequently starved in medium without FBS for 2 h. Autophagosomes are indicated by 18:1 NBD-PS signal not co-localizing with mitochondria [26].

### 2.12. Statistical Analysis

Statistical significance was determined with GraphPad Prism 8.0 software. Statistical tests and number of replicates are indicated in detail in the figure legend for each experiment. We used nonparametric tests in order to account for the small sample size. All experiments were repeated at least three times (the number of independent biological replicates is indicated by *n*).

## 3. Results

### 3.1. Identification of the Novel Mutations T351A and T610A in the RHOT1 Gene in PD Patients

**Figure 1:**
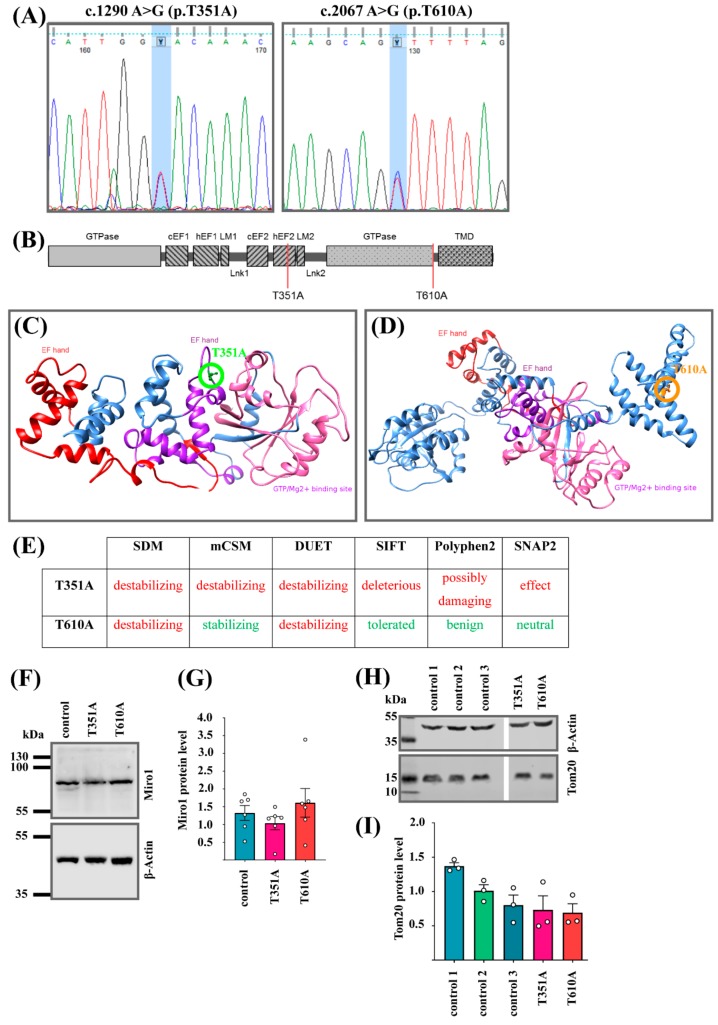
(**A**) Sanger sequencing result for the mutations c.1290A > G and c.2067A > G (NM_001033568), leading to the amino acid exchanges T351A or T610A (NP_001028740), respectively. (**B**) Schematic overview of Miro1 protein structure, showing the two newly identified mutations in Miro1: T351A is located within the second EF-hand domain and T610A within the C-terminus. (**C**) Homology model of human Miro1 based on the 3D structure of *Drosophila* Miro. The 3D structure shows both EF-hand domains, the C-terminal GTPase domain and the C-terminus. The amino acid T351 is highlighted with a green circle, (**D**) while the amino acid position T610 is highlighted with an orange circle. (**E**) Overview of the mutation effects on protein stability and functionality as predicted by SDM, mCSB, DUET, SIFT, Polyphen2, and SNAP2. (Red is “destabilizing”, “deleterious” or “possibly damaging”; Green is “stabilizing”, “tolerated” or “benign”. As indicated in the table.) (**F**) Representative Western blot image of Miro1 protein in immortalized fibroblasts with the mutations Miro1-T351A or Miro1-T610A. (**G**) Densitometry of Western blot analysis from (F) for Miro1 protein levels normalized to β-Actin. Data indicated as mean ± SEM (*n* = 6). (**H**) Representative Western blot image of Tom20 protein in immortalized fibroblasts. (**I**) Densitometry of Western blot analysis of Tom20 protein levels normalized to β-Actin. Data indicated as mean ± SEM (*n* = 3).

Recently, we identified the first disease-associated mutations in *RHOT1* in two German PD patients [10]. Here, we describe two additional *RHOT1* mutations found in two male PD patients of German origin. The heterozygous point mutations c.1290A > G and c.2067A > G (NM_001033568) leading to the amino acid exchanges T351A or T610A (NP_001028740), were validated by Sanger sequencing (Figure 1A).

Whole exome sequencing excluded mutations in other known PD genes in these patients. The mutation T351A is located within the second calcium-binding EF-hand domain, while the mutation T610A is located within the C-terminus of Miro1 (Figure 1B). Both affected amino acids are exposed to the cytosol at the surface of the protein (Figure 1C,D).

For the T351A mutation, all effect prediction algorithms consistently estimate a destabilizing effect or, respectively, a “deleterious” or “possibly damaging” effect. For the T610A mutation, different methods estimate different effects, with two approaches (SDM and DUET) predicting a destabilizing effect, and the other methods predicting a stabilizing effect (mCSM), benign or neutral effect (Polyphen2, SNAP2), or a toleration of the mutation (but with low confidence, SIFT; Figure 1E).

The lower confidence and disagreement between the predictions in this case is not surprising, given that the T610 residue was only covered by the lower-quality homology model for Miro1. Both threonine residues in the native structure are highly conserved, and in both cases the mutated residue is smaller and more hydrophobic than the wild-type residue. However, the T351 residue is exposed on the protein surface, whereas only a medium exposure is predicted for the T610 residue (prediction by RaptorX software).

Interestingly, using the further dedicated software tool MutationTaster [27] to assess possible effects on transmembrane domains (TMD), the T610A mutation is predicted to result in the loss of a TMD close to the C-terminal end of the protein structure. Thus, while the T351A mutation is most likely causing a destabilization of the Miro1 protein structure, the T610A mutation may rather only affect the surrounding structural region, possibly including the nearby TMD (Figure 1E).

Western blot analysis revealed that the relative amount of Miro1 protein was not affected in patient-derived mutant fibroblasts compared to control cells (Figure 1F,G). Furthermore, the relative amount of the mitochondrial marker protein Tom20 was comparable between both mutant fibroblasts lines and three control fibroblast lines (Figure 1H,I).

### 3.2. Increased Calcium Stress is a Shared Phenotype Across Different Miro1 Mutations in PD

**Figure 2:**
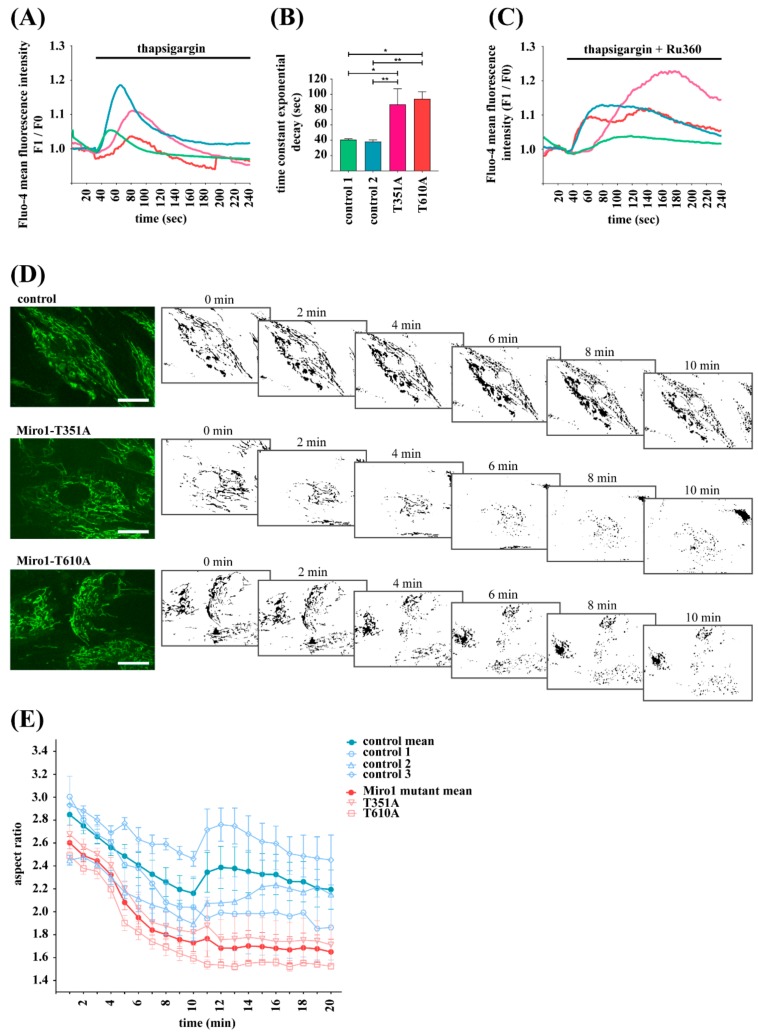
(**A**) Immortalized fibroblasts were loaded with the cytosolic calcium indicator Fluo-4 AM for live cell imaging. During imaging, cells were treated with 1 µM thapsigargin in order to inhibit calcium uptake by the SERCA pumps and to deplete endoplasmic reticulum (ER) calcium stores. Imaging was continued for 10 min with a 2 s interval. Images were obtained with a 25× objective. Data indicate the fluorescence signal intensity of Fluo-4 AM expressed as mean fluorescence F1/F0. (**B**) Time constant of the exponential decay calculated from the calcium response curves from (A). The data indicate the time, which is needed to recover from the thapsigargin-induced cytosolic calcium peak shown in (A). Data indicated as mean ± SEM. Significance calculated by Mann-Whitney test (*n* = 4). * *p* < 0.05; **: *p* < 0.001. (**C**) Immortalized fibroblasts were loaded with Fluo-4 AM for live cell imaging and treated with 1 µM thapsigargin and 10 µM Ru360 in order to inhibit calcium buffering by the ER and by the mitochondrial calcium uniporter (MCU). Cells were imaged for 10 min with a 2 sec interval using a 25× objective. Data is expressed as mean Fluo-4 AM fluorescence intensity F1/F0 (*n* = 3). (**D**) Immortalized fibroblasts were stained with MitoTracker green FM for live cell imaging. Images were obtained once per minute using a 40× objective. During imaging, cells were treated with 20 µM ionomycin and mitochondrial morphology was analyzed using ImageJ. Mitochondrial masks from image analysis are shown for all cell lines at different time points. Scale bars indicate 20 µm. (**E**) Analysis of mitochondrial fragmentation in different fibroblast lines, expressed as aspect ratio, from images shown in (D); (*n* = 3–5).

Miro1 acts as sensor for cytosolic calcium levels [8,28,29]. Accordingly, we recently showed that patient-derived fibroblasts expressing mutant Miro1 proteins display a decreased capacity to buffer cytosolic calcium after inhibition of ER calcium ATPase by thapsigargin treatment [10]. In the present study, we stained fibroblasts with the cytosolic calcium indicator Fluo4-AM and treated cells during live cell imaging with thapsigargin. In all investigated fibroblast cultures, thapsigargin induced a fast increase of cytosolic calcium levels by inhibition of ER-mediated calcium uptake and depletion of ER calcium stores [30] (Figure 2A). However, calculating the time constant of the exponential decay from the calcium response curve revealed that control cells recover considerably faster from this peak than both Miro1-mutant fibroblasts (Figure 2B).

In order to assess the contribution of mitochondria to the buffering of cytosolic calcium after thapsigargin treatment, we treated fibroblasts in parallel with Ru360, an inhibitor of the mitochondrial calcium uniporter (MCU [28,31,32]). The resulting response curve indicates that blockage of mitochondrial calcium import abolishes the ability of all cell lines to compensate for elevated cytosolic calcium (Figure 2C). This finding suggests that calcium buffering in fibroblasts after thapsigargin treatment is mainly facilitated by mitochondria.

Cytosolic calcium was shown to regulate mitochondrial morphology, with increasing calcium levels causing fragmentation [8]. In the light of this result, we were interested in investigating mitochondrial morphology in response to calcium stress. For this purpose, fibroblasts were stained with MitoTracker green FM and treated with the calcium ionophore ionomycin during live cell imaging. Quantifying the mitochondrial aspect ratios after ionomycin exposure, we observed calcium-mediated mitochondrial fragmentation in all fibroblast lines. By contrast, this fragmentation process was faster and stronger in both Miro1-mutant fibroblast lines compared to controls (Figure 2D,E). These results are in line with our findings in Miro1-R272Q and Miro1-R450C fibroblasts [10], suggesting that all four identified PD-associated Miro1 variants cause a similar calcium phenotype.

### 3.3. Mutations in Miro1 Cause Reduction of MERCs

**Figure 3:**
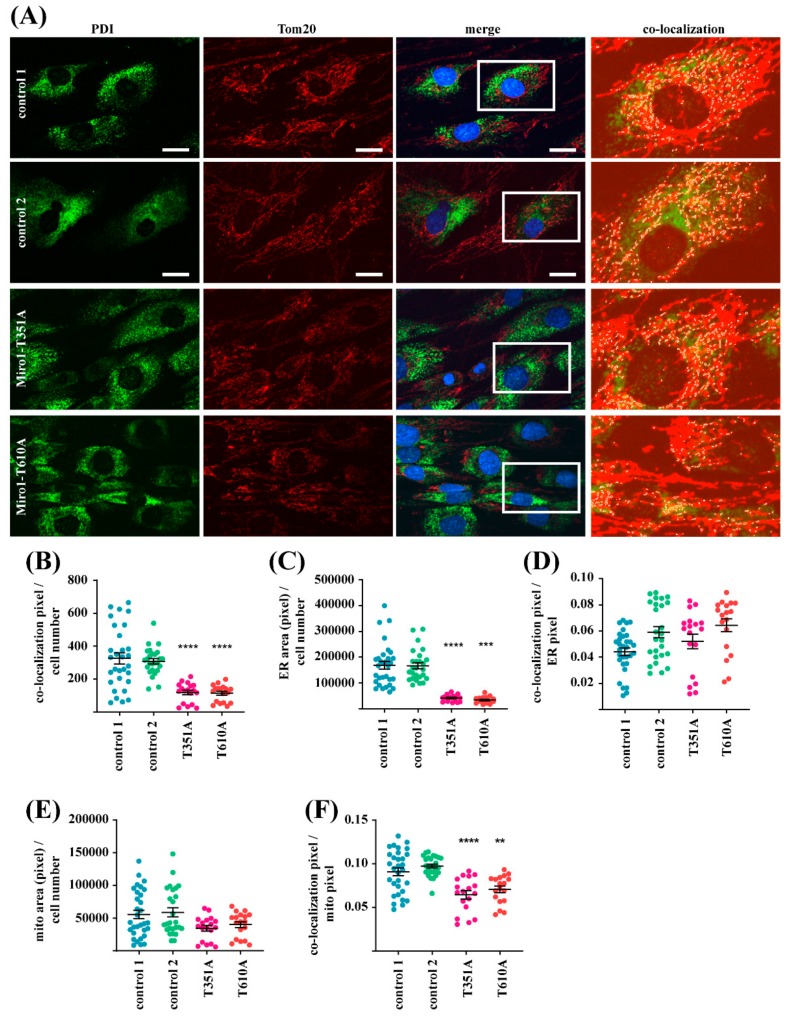
(**A**) Native fibroblasts were fixed and stained with antibodies against the ER marker protein protein disulfide-isomerase (PDI) and the mitochondrial marker protein Tom20. Images were obtained using a 63× objectives; scale bars indicate 20 µm. The white boxes in the merged images indicate the magnified regions shown in the co-localization panels. Co-localization of PDI and Tom20 signals was analyzed with MatLab. Co-localization events are highlighted as white dots. (**B**) Quantification of co-localization events of PDI and Tom20 per cell, indicating the amount of mitochondrial-endoplasmic reticulum contact sites (MERCs). (**C**) Quantification of ER area per cell in pixel from the PDI signal. (**D**) Quantification of co-localization events of PDI and Tom20 normalized to PDI-positive ER pixel. (**E**) Quantification of mitochondrial area per cell from the Tom20 signal. (**F**) Quantification of co-localization events of PDI and Tom20 per mitochondrial area. All data indicated as mean ± SEM. Significance calculated using a Kruskal Wallis test (*n* = 3; 25 cells analyzed per fibroblast line per experiment). ** *p* < 0.001; *** *p* < 0.0001; **** *p* < 0.00001.

Several previous studies showed that cellular calcium buffering depends on MERCs and that their regulation is mediated by Miro1 [5,8]. We stained fixed fibroblasts with antibodies against the ER marker PDI and the mitochondrial marker Tom20 in order to analyze the co-localization of both organelles (Figure 3A). This analysis showed that the amount of MERCs, indicated by the number of co-localization events, was reduced in both Miro1-mutant fibroblast lines, compared to controls (Figure 3B).

Furthermore, quantification of the PDI signal revealed a significantly reduced ER area per cell, which is indicative of ER mass (Figure 3C). When normalized to ER area, the amount of MERCs was not different between controls and mutant fibroblasts (Figure 3D).

We also assessed the mitochondrial mass from the Tom20 signal and found no statistical significant difference between controls and mutants (Figure 3E). The amount of MERCs normalized to mitochondrial area was also significantly reduced in T351A and T610A fibroblasts (Figure 3F). These phenotypes are in line with the observation of reduced MERCs and reduced ER mass in the previously described PD-associated Miro1 mutants R272Q and R450C [10].

### 3.4. Reduced Localization of Mutant Miro1 to MERCs

**Figure 4:**
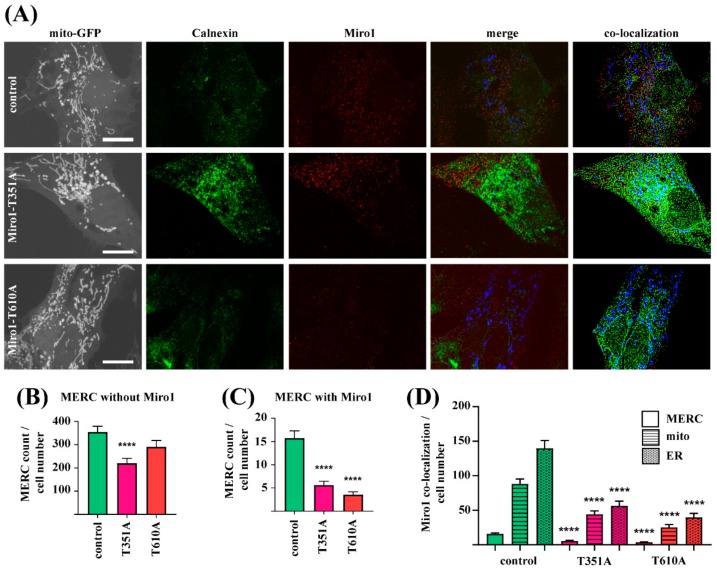
(**A**) Immortalized fibroblasts were transfected with mito-GFP and fixed 24 h post-transfection for subsequent labeling with antibodies against Miro1 and the ER marker Calnexin. Images were obtained using a 63× objective; scale bars indicate 20 µm. Co-localization events were analyzed using MatLab. (**B**) Quantification of MERCs without Miro1 and (**C**) MERCs with Miro1 per cell from images shown in (A). (**D**) Quantification of co-localization events of Miro1 puncta with MERCs, mitochondria or the ER per cell. All data indicated as mean ± SEM. Significance calculated with Kruskal-Wallis test (*n* = 3; ~20 cells analyzed per fibroblast line per experiment). **** *p* < 0.00001.

Since Miro1 is a crucial regulator of MERCs, we were interested to assess whether different mutations in Miro1 have an impact on the proteins localization to MERCs. For this purpose, fibroblasts were first transfected with mito-GFP and afterwards fixed for antibody staining with the ER protein calnexin and Miro1 (Figure 4A). Co-localization analysis showed that Miro1-T351A, but not T610A fibroblasts also present less MERCs without Miro1, compared to control fibroblasts (Figure 4B). Of note, in all fibroblast lines only a subset of MERCs stained positive for Miro1 (Figure 4C), and the mutant T351A and T610A fibroblasts display significantly less MERCs with Miro1 compared to control fibroblasts (Figure 4C).

These results suggest that the observed overall reduction of MERCs in Miro1-mutant fibroblasts is might be driven by a reduction of Miro1-containing MERCs. In line, further image analyses revealed, that all mutant fibroblast lines show a reduction of Miro1 localization to mitochondria and to the ER (Figure 4D).

### 3.5. Mutations in Miro1 Cause Alterations of MERC Types

**Figure 5:**
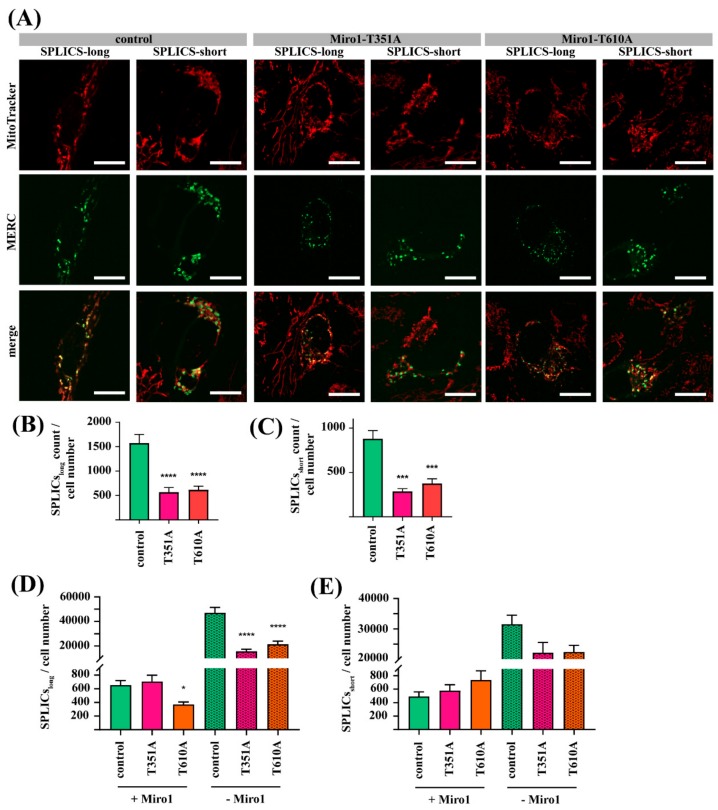
(**A**) Immortalized fibroblasts were transfected either with the SPLICS-long, or with the SPLICS-short construct. After 12 h, cells were stained with MitoTracker deep red FM for live cell imaging, using a 63x objective; scale bars indicate 20 µm. (**B**) Quantification of wide MERCs (from SPLICS-long signal) and (**C**) narrow MERCs (from SPLICS-short signal) per cell. Fibroblasts transfected with (**D**) SPLICS-long or (**E**) SPLICS-short constructs were fixed and stained with an antibody against Miro1. Afterwards, cells were imaged with a 63× objective and SPLICS signals with and without Miro1 were quantified. All data indicated as mean ± SEM. Significance was assessed using a Kruskal-Wallis test (*n* = 3; ~16 cells analyzed per fibroblast line per experiment). * *p* < 0.05; *** *p* < 0.0001; **** *p* < 0.00001.

To further elucidate the quality of MERCs, we transfected fibroblasts with so-called split-GFP-based contact site sensor (SPLICS) constructs [23]. These constructs express two parts of a split GFP protein in the same plasmid, one part being targeted to the ER and the second part targeted to the mitochondria. As soon as both organelles come in close proximity, the mitochondrial and the ER part of the split GFP align and give a fluorescent signal. While the SPLICS-short construct labels narrow MERCs with a distance between ER and mitochondria of 8–10 nm, the SPLICS-long construct labels wide MERCs with a distance of 40–50 nm between both organelles [33]. Transfected fibroblasts were stained with MitoTracker deep red and live cell imaging showed that the SPLICS signal co-localized with mitochondria (Figure 5A).

Quantification of SPLICS signal per cell revealed that all fibroblasts, controls and mutant lines, have in general more wide MERCs (SPLICS-long) than narrow MERCs (SPLICS-short; Figure 5B,C), a result that fits to previous observations [33]. Both Miro1-mutant fibroblasts display significantly less wide (Figure 5B) and narrow (Figure 5C) MERCs compared to control fibroblasts. These results suggest that mutations in Miro1 cause changes in the structure of MERCs.

Fibroblasts transfected with SPLICS constructs were fixed and stained with an antibody against Miro1 for subsequent quantification of SPLICS signals with Miro1 or without Miro1, respectively. Only the T610A mutant shows a reduction of SPLICS-long signal with Miro1 (Figure 5D), while both mutant fibroblast lines, T351A and T610A, show a significant reduction of SPLICS-long signal without Miro1 co-localization (Figure 5D). In contrast, both mutant fibroblast lines do not show changes of SPLICS-short signal with Miro1 and a slight reduction of SPLICS-short signal without Miro1 (Figure 5E). These findings support our previous observation that Miro1 mutations cause alterations of the structure of MERCs.

### 3.6. LC3-Dependent Autophagy is Impaired in Miro1-T351A and Miro1-T610A Fibroblasts

**Figure 6:**
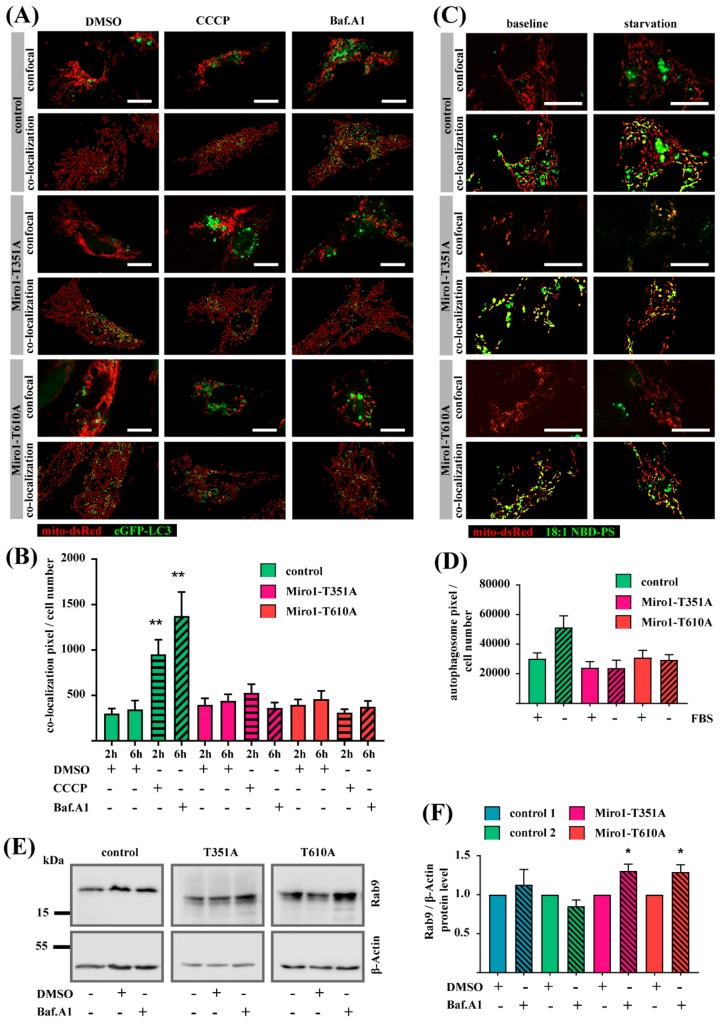
(**A**) Fibroblasts were transfected with mito-DsRed and eGFP-LC3 and treated with 25 µM CCCP for 2 h or with 10 nM BafilomycinA1 for 6 h. Microscopy images were obtained with a 40× objective. In the co-localization panel, the mitochondria are indicated in green and the LC3 puncta are indicated in red. Co-localization events of mitochondria and LC3 puncta are indicated in yellow. Scale bars indicate 20 µm. (**B**) Quantification of co-localization events of mitochondria and LC3 puncta from (A) normalized to cell number. Significance was calculated using the Mann-Whitney test (*n* = 3; ~24 cells analyzed per fibroblast line, per condition and per experiment). (**C**) Fibroblasts were transfected with mito-DsRed and after 24 h loaded with 18:1 NBD-PS. Fibroblasts were then starved without FBS for 2 h before live cell imaging, using a 63× objective. The co-localization panel shows mitochondria in red and 18:1 NBD-PS signal in green. Co-localization of mitochondria and 18:1 NBD-PS is indicated in yellow. Autophagosomes were identified as 18:1 NBD-PS signal, which did not co-localize with the mito-DsRed signal. (**D**) Quantification of autophagosomes from microscopy images shown in (C). Significance calculated with Mann-Whitney test (*n* = 3; 10 cells analyzed per line, condition and per experiment). (**E**) Western blot image of Rab9 and β-Actin in immortalized fibroblasts. (**F**) Quantification of relative Rab9 protein levels normalized to β-Actin. Significance calculated by Mann-Whitney test (*n* = 4–6). All data indicated as mean ± SEM. * *p* < 0.05; ** *p* < 0.001.

MERCs play a crucial role not only in calcium homeostasis, but also in the regulation of mitophagy [8]. In our previous study describing the Miro1 mutations R272Q and R450C, we found changes in mitochondrial quality control mechanisms in patient-derived fibroblasts. Hence, we investigated autophagy pathways in the T351A and T610A fibroblasts. Fibroblasts were transfected with mito-dsRed and eGFP-LC3 for subsequent treatment with the mitochondrial uncoupler CCCP or Bafilomycin A1, an inhibitor of lysosomal degradation (Figure 6A). Co-localization of mitochondria with LC3 puncta indicated mitophagy. In control fibroblasts, CCCP treatment leads to an increased co-localization of mitochondria and LC3 puncta, indicating increased mitophagy. Inhibition of lysosomal degradation by Bafilomycin A1 also leads to an increase of co-localization events in control fibroblasts. In contrast, neither of the treatments results in changes of mitochondria-LC3 co-localization in T351A- or T610A-fibroblasts (Figure 6B).

This result points to an inhibition of the autophagy flux in these mutant lines. Therefore, we were interested in further analysis of the LC3-dependent autophagy pathway. Formation of LC3-dependent autophagosomes requires transfer of the lipids phosphatidylethanolamine (PE) and phosphatidylserine (PS) at MERCs [34]. PS is transformed into PE and together with LC3 gets integrated into the ER membrane to form autophagosomes. Therefore, the translocation of the 18:1 NBD-PS signal from mitochondria to the cytosol serves as readout for autophagosome formation [8,10,26]. We loaded fibroblasts with the fluorescent labeled lipid 18:1 NBD-PS and starved them without FBS to induce LC3/ATG5-dependent autophagy [34]. Afterwards, we quantified the 18:1 NBD-PS signal, which was not co-localized with mito-dsRed-labeled mitochondria (Figure 6C). In control fibroblasts, FBS starvation induced autophagosome formation, while both mutant cell lines showed no effect on autophagosome number under stress conditions (Figure 6D), indicating an inhibition of autophagosome formation.

In our previous study we showed that Miro1-mutant fibroblasts with the mutations R272Q or R450C displayed an increased lysosomal turnover of Rab9 [10], a marker protein for ATG5 / LC3-independent autophagy. Within this pathway, autophagosomes are not derived from the ER membrane at MERCs, but derive from the Golgi apparatus, mediated by Rab9 [35]. Hence, we quantified Rab9 protein levels under Bafilomycin A1 treatment. Interestingly, Bafilomycin A1 treatment has no effect on Rab9 levels in control fibroblasts, but increased Rab9 in both mutant lines (Figure 6E,F). Together, these results suggest that T351A and T610A mutant fibroblasts might use the Rab9-dependent autophagy pathway while the LC3-dependent pathway is impaired.

## 4. Discussion

Miro1 is a crucial sensor for cytosolic calcium levels and is furthermore involved in the regulation of calcium homeostasis at the contact sites between mitochondria and the ER. MERCs are an emerging topic in the field of PD research and a number of PD-associated proteins were found to be involved in the regulation of MERCs, i.e., PINK1, Parkin, and LRRK2 [5,6,7,36]. Already in 2011, Kornmann and colleagues showed that Miro1 plays a role at the contact sites in mammalian Cos-7 cells [37]. Moreover, in yeast cells devoid of the Miro1 orthologue Gem1, the number of MERCs was significantly decreased [37]. This confirms our previous observation of reduced MERCs in the Miro1 mutants R272Q and R450C, which also displayed a reduced amount of total Miro1 protein [10]. However, while the two mutations T351A and T610A described in the current study did not show reduced Miro1 protein levels in fibroblasts, a decrease in the number of MERCs and impaired calcium homeostasis is suggested to be a shared hallmark across all currently known PD-associated Miro1 mutations.

In addition to altered MERC numbers and calcium dyshomeostasis, we showed here that there is a shift in the proportion of wide and narrow MERCs in patient-derived fibroblasts. First, it is worth noting that in all fibroblast lines (independent of the genotype) the majority of MERCs were devoid of Miro1. This observation is in line with previous studies showing that Miro1 acts as a regulatory protein at the MERCs but is not required for the assembly of MERCs [37]. However, all four mutant fibroblast lines, R272Q, R450C (Supplementary data 1A–C), T351A, and T610A contained significantly fewer Miro1-positive MERCs compared to control fibroblasts. Furthermore, co-localization analyses revealed that also less Miro1 was recruited to mitochondria and the ER compartments compared to control cells in all four Miro1 mutants (Supplementary Data 1D). These results raise the question whether the mutations in Miro1 interfere with the localization of Miro1 to MERCs and destabilize the contact sites between mitochondria and ER.

Electron microscopy revealed that MERCs are formed with varying distances between ER and mitochondria [38]. Different mechanisms regulate the distance of the cleft between ER and mitochondria at MERCs suggesting that wide and narrow MERCs are involved in distinct cellular processes. While the smooth ER forms connections with mitochondria at a distance of 8–10 nm, the rough ER forms mitochondria connections at 50–60 nm [39]. Moreover, the distance between ER and mitochondria membranes at MERCs changes as a result of metabolic alterations. Starvation increased the distance of MERCs and over-nutrition decreased the distance in mice liver [38,40].

With the SPLICS method, Cieri and colleagues could additionally show that the distance of MERCs is regulated during processes such as ER stress, mitochondrial elongation, fragmentation, and mitophagy [33]. Earlier studies showed that MERCs, which facilitate calcium transfer between ER and mitochondria via a complex of IP3R, Grp75, and VDAC [41], have a cleft width of ~15–20 nm [42,43]. MERC clefts that are narrower than 7 nm or wider than 25 nm inhibit the formation of the protein complex required for calcium transfer [38,40,44]. Accordingly, the SPLICS-short signal, which visualizes MERCs with a cleft of ~10 nm [33], is indicative of effective calcium transfer between ER and mitochondria. From our results we therefore conclude that the impaired calcium homeostasis could result from the reduction of narrow MERCs that was consistently observed in all four Miro1-mutant fibroblast lines (Supplementary Data 2C).

The SPLICS-long construct visualizes MERCs with a width of ~50 nm [33], which have been associated with the formation of LC3/ATG5-dependent autophagosomes [34]. The Miro1 mutations R272Q and R450C do not display a reduction of these wide MERCs (Supplementary Data 2B), which is in line with our previous observation that autophagosome formation and LC3-dependent mitophagy is indeed increased in fibroblasts with those mutations [10]. In contrast, the mutations T351A and T610A show a considerable decrease of wide MERCs alongside an impaired flux of LC3-dependent autophagy. It is tempting to speculate that the differences of wide and narrow MERCs observed in the R272Q and R450C, and the T351A and T610A mutations are related to the different mitophagy phenotypes in both sets of mutations. Differential effects of mutations have previously also been observed in fibroblasts derived from patients with monogenic PD [45]. This phenomenon warrants further investigation in neuronal models with Miro1 mutations.

However, co-localization analysis of SPLICS with Miro1 revealed that especially the SPLICS-long signal without Miro1 puncta was significantly reduced in T351A and T610A mutant fibroblasts. This result raised the question how mutant Miro1 could interfere with the amount of Miro1-negative MERCS. MERCS represented by SPLICS-long signals were previously associated with mitophagy. Conditions, which facilitate mitophagy, e.g., tunicamycin treatment, knockdown of Mfn2, activation of Parkin or Drp1-induced mitochondrial fragmentation reduced the amount of SPLICS-long signal [33]. Therefore, one could assume that the observed alterations of the mitophagy pathway are not only affecting MERCS containing Miro1, but alter MERCS architecture in general.

With the identification and functional characterization of two novel mutations in Miro1, we provided further evidence for *RHOT1* as a risk gene for PD. Moreover, our observation of altered abundance and composition of MERCs further emphasizes their critical involvement in the pathogenesis of PD.

## Supplementary Materials

The following are available online at https://www.mdpi.com/2077-0383/8/12/2226/s1, Supplementary data 1: Reduced localization of mutant Miro1-R272Q and Miro1-R450C to MERCs; Supplementary data 2: Miro1-R272Q and Miro1-R450C cause alterations of MERC types
