# Supplementary material for: Variants in Miro1 Cause Alterations of ER-Mitochondria Contact Sites in Fibroblasts from Parkinson’s Disease Patients
Source: J Clin Med. 2019 Dec 16;8(12):2226. doi: 10.3390/jcm8122226 (PMC6947516; doi:10.3390/jcm8122226)
Supplement: Supplementary file 1 [file jcm-08-02226-s001.pdf]

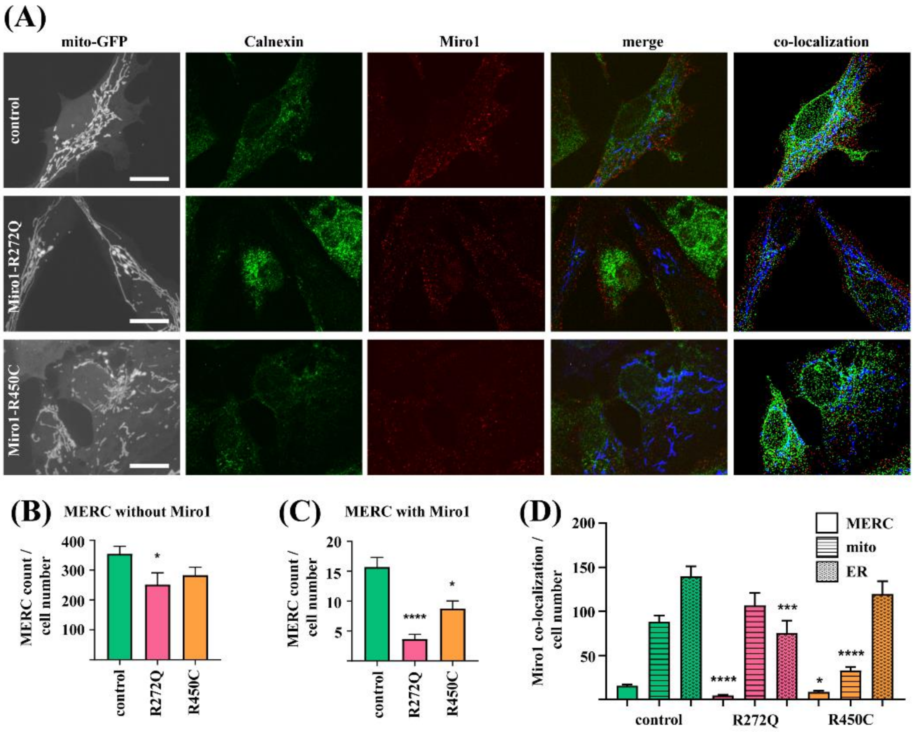

Supplementary data 1: *Reduced localization of mutant Miro1-R272Q and Miro1-R450C to MERCs*
(A) Immortalized fibroblasts were transfected with mito-GFP. 24 hours after transfection cells were fixed and labeled with antibodies against Miro1 and the ER marker Calnexin. Images were obtained using a 63x objective; scale bars indicate 20 µm. Co-localization events were analyzed using MatLab. **(B)** Quantification of MERCs without Miro1 and **(C)** MERCs with Miro1 per cell. **(D)** Quantification of co-localization events of Miro1 with MERCs, mitochondria or the ER per cell. All data indicated as mean ± SEM. Significance calculated with Kruskal-Wallis test (n=3). * p<0.05; ** p<0.001; *** p<0.0001; **** p<0.00001.

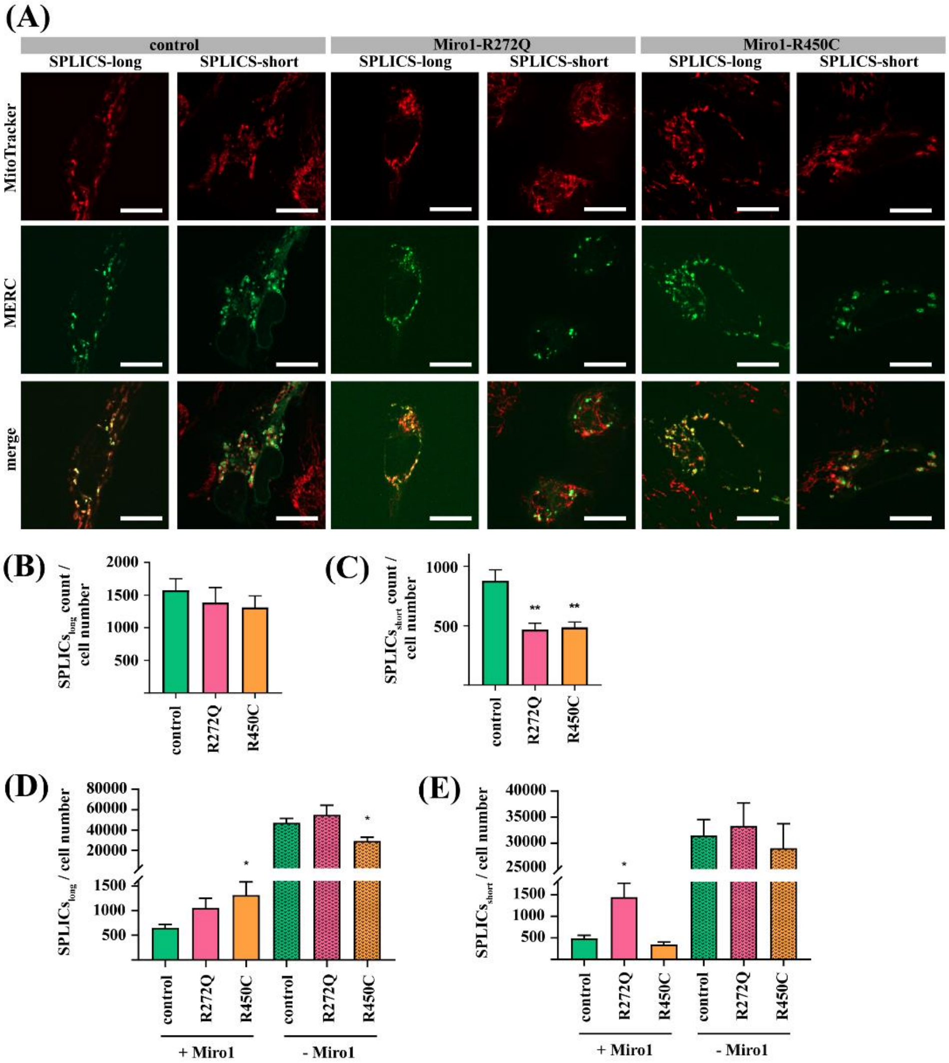

Supplementary data 2: *Miro1-R272Q and Miro1-R450C cause alterations of MERC types*
(A) Immortalized fibroblasts were transfected with the SPLICS-long, or with the SPLICS-short construct and incubated for 12 hours. Then, cells were stained with MitoTracker deep red for live cell imaging, using a 63x objective. Scale bars indicate 20 µm. **(B)** Quantification of SPLICS-long signal and **(C)** SPLICS-short signal per cell. Fibroblasts were transfected with **(D)** SPLICS-long or **(E)** SPLICS-short constructs and afterwards fixed and stained with a Miro1-antibody for imaging, using a 63x objective. Afterwards, Miro1-positive and Miro1-negative SPLICS signals were quantified. All data indicated as mean ± SEM. Significance was assessed using a Kruskal-Wallis test (n=3). * $p<0.05$; ** $p<0.001$; *** $p<0.0001$; **** $p<0.00001$.
